# Phase II study of docetaxel in patients with relapsed or refractory malignant lymphoma

**DOI:** 10.1038/sj.bjc.6600914

**Published:** 2003-04-29

**Authors:** J M Zekri, R E Hough, J M Davies, R Molife, B W Hancock, P C Lorigan

**Affiliations:** 1 Cancer Research Centre, Weston Park Hospital, Whitham Road, Sheffield S10 2SJ, UK; 2 Haematology Department, The Royal Hallamshire Hospital, Sheffield S10 2JF, UK; 3 Western General Hospital, Crewe Road, Edinburgh EH3 2XU, UK; 4 Oncology Department, Nottingham City Hospital, Hucknall Road, Nottingham NG5 1PB, UK

**Keywords:** docetaxel, non-Hodgkin's lymphoma, Hodgkin's disease

## Abstract

We report the activity and toxicity of docetaxel in 12 evaluable heavily pretreated patients with relapsed and refractory non-Hodgkin's lymphoma and Hodgkin's disease. In all, 42% achieved a partial response, 25% achieved stable disease. Median duration of response was 16 (10–21) weeks. The median overall survival was 70 (9–178) weeks and for responders it was 120 (22–178) weeks. One patient developed one episode of neutropenic sepsis. Docetaxel has limited activity in this group of patients.

Patients with refractory/relapsed non-Hodgkin's lymphoma (NHL) and Hodgkin's disease (HD), who are not suitable for high-dose chemotherapy (HDC) or who have relapsed after HDC, have a poor prognosis with currently available salvage chemotherapy regimens (El Helw *et al*, 2000; Shamash *et al*, 2000). New approaches are required to improve the prognosis of these patients.

The taxanes (paclitaxel and docetaxel) are a novel group of cytotoxic compounds with a broad spectrum antitumour activity and have different mechanisms of action from other chemotherapeutic agents. They exert their cytotoxic effect by inhibiting microtubular depolymerisation (Horwitz *et al*, 1994); with docetaxel being more potent than paclitaxel (Ringel and Horwitz, 1991). Docetaxel has antiapoptotic and antiangiogenic properties that may contribute to cytotoxic activity (Haldar *et al*, 1997; Hortobagyi, 1999).

Docetaxel has shown significant activity in breast cancer, nonsmall cell lung cancer and ovarian cancer. (Crown, 1999; Gandara *et al*, 1999; Vasey *et al*, 2001; Markman *et al*, 2001).

In previous studies; paclitaxel showed some activity in relapsed/refractory NHL and HD (Wilson *et al*, 1995; Hopfinger *et al*, 1996; Younes *et al*, 1997, Younes *et al*, 2001). The experience in the UK with docetaxel in lymphoma is limited. However, because of its mode of action, activity in other solid tumours and convenience of administration, docetaxel is a potentially attractive candidate for evaluation in NHL and HD.

We report a phase II study of docetaxel in patients with relapsed and refractory NHL and HD.

## PATIENTS AND METHODS

In total, 13 patients recruited into the study had histologically proven, relapsed or refractory NHL or HD for whom further conventional chemotherapy was felt to be of little potential benefit. Patients were excluded if they had central nervous system lymphomatous involvement. All patients gave informed consent in accordance with research ethics committee approval. In all, 13 patients were recruited into this trial; their clinical characteristics are listed in Table 1

**Table 1:** Individual patients' characteristics, previous treatment, study treatment and response

						**Response after cycle**					
**Sex**	**Age (years) mean =51**	**No of previous chemotherapy regimens**	**Histology**	**Starting dose (mg m^−2^)**	**No. of cycles**	**2**	**4**	**6**	**DOR in PR PFS in SD+PR (weeks)**	**Patient status**	**Survival (weeks)**
M	61	1	HG NHL^a^	100	5	PR	PR	PD (After 5)	16	Dead	125
F	53	2	HG NHL^b^	100	4	PR	PD	—	12	Dead	120
M	51	3 (HDC)	HG NHL^a^	100	1	PD (After 1)	—	—		Dead	9
M	48	3 (HDC)	HG NHL^a^	100	1	NE	—	—	NE	Dead	15
M	55	4 (HDC)	HG NHL^b^	100	3	SD	PD (After 3)	—	9	Dead	70
M	57	3	HG NHL^a^	100	6	SD	SD	SD	30	Dead	53
F	56	5 (HDC)	HG NHL^a^	70	2	PD	—	—		Alive	15
F	42	6 (HDCx2)	LG NHL^a^	100	2	PR	—	—	10	Dead	70
M	59	5	LG NHL^b^	70	6	SD	SD	SD	20	Alive	21
F	47	2	HD^a^	100	4	PR	PD	—	17	Alive	178
F	27	3 (HDC)	HD^a^	100	2	PD	—	—		Alive	144
M	53	3 (HDC)	HD^a^	100	6	PR	PR	PR	21	Dead	22
F	57	3 (HDC)	HD^a^	100	2	PD	—	—		Dead	35

HD=Hodgkin's disease, NHL=non-Hodgkin's lymphoma, LG=low grade, HG=high grade, M=male, F=female, DOR=duration of response, PFS=progression-free survival,

a =relapsed,

b =refractory, NE=not evaluable, HDC=high dose chemotherapy.

.

Patients were premedicated with dexamethasone 8 mg orally 13 h and 1 h prior to docetaxel infusion. The antiemetic regimen was dexamethasone 8 mg and granisteron 3 mg i.v. before chemotherapy and dexamethasone 8 mg po bd for 4 days following each cycle. Docetaxel 100 mg m^−2^ was infused intravenously. Treatment was repeated every 3 weeks in an outpatient setting. In response to the findings by other authors that reducing the dose of docetaxel significantly reduced grade IV neutropenia and febrile neutropenia in breast and lung cancer patients (Salminen *et al*, 1999; Fossella *et al*, 2000), the study protocol was amended and the final two patients received 70 mg m^−2^.

Patients were evaluated for treatment response after every second cycle according to WHO standard criteria. Repeat response confirmation scans were not performed. Patients with complete response (CR), partial response (PR) or stable disease (SD) were continued on treatment for up to six cycles. Patients who had progressive disease (PD) after any cycle were withdrawn from the study. A full blood count and biochemistry profile were carried out weekly.

Dose modification and delay was permitted in the event of some treatment-related side effects.

## STATISTICS

The duration of response dates from beginning of treatment until evidence of progression. The progression free survival dates from beginning of treatment until evidence of progression in those who achieved PR and SD. Overall survival dates from the start of treatment until death or last follow-up if alive.

SPSS software package was used for statistical analysis. Progression-free survival and overall survival were analysed using the Kaplan–Meier curves.

## RESULTS

### Response to treatment

Table 1 summarises the patient's characteristics and response to docetaxel. Overall, 12 patients were evaluable for response after any number of cycles (one to six). There were no complete responders. After the first two cycles five out of 12 (42%) had PR. After four cycles, this response was maintained in two patients (17%). Three (25%) patients had SD after the second cycle of treatment.

Three out of 12 patients had disease refractory to previous chemotherapy, one had had PR and the other two patients had SD after docetaxel treatment. Nine patients had relapsed disease; four (44%) achieved PR. Four patients had had CR to their previous chemotherapy; only two out of four achieved PR to docetaxel, but lasting only for two cycles of therapy.

**Figure 1:**
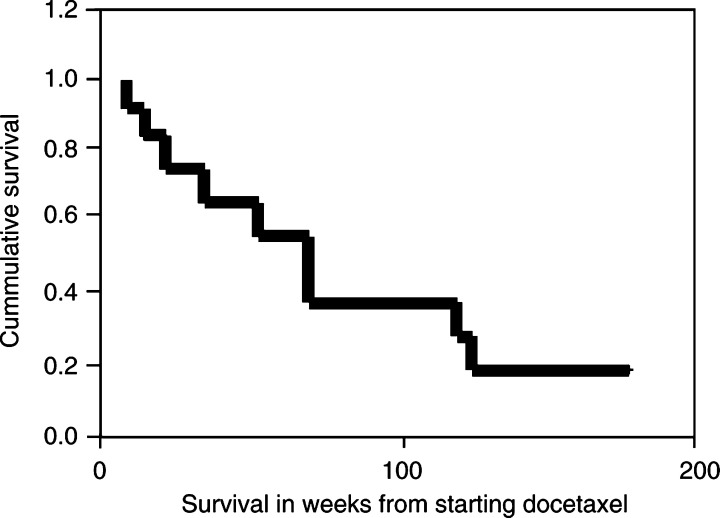
Overall survival (*n*=13).

**Figure 2:**
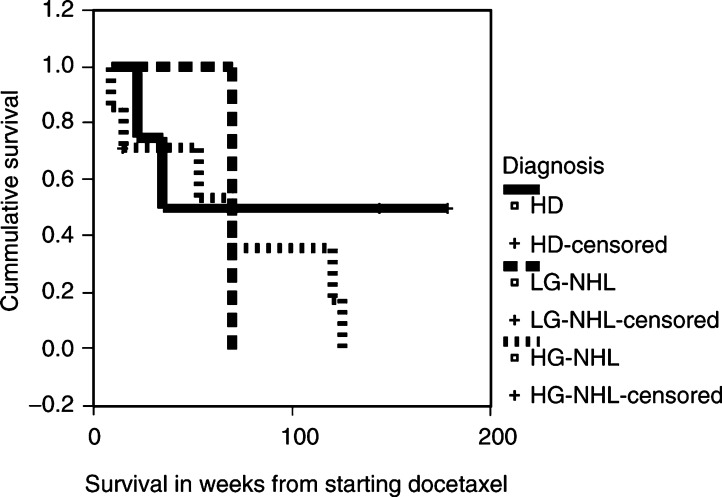
Survival of diagnoses groups.

The median overall survival for all patients was 70 (9–178) weeks (Figure 1

). The median survival for patients with HD was 35 (22–178), HG-NHL 70 (9–125) and for LG-NHL 70 (21–70) weeks (Figure 2

).

The median duration of response was 16 (10–21) weeks and the median survival 120 (22–178) weeks for responders. Patients achieving PR or SD had a median progression-free survival of 16.5 (9–30) weeks and median survival of 70 (21–178) weeks.

Four patients had Hodgkin's disease, two progressed after two cycles of docetaxel, while two achieved PR. Six patients had HG-NHL; two, two and two had PR, SD and PD, respectively. Two patients had LG-NHL; one had PR and one had SD. seven out of 12 (58%) evaluable patients had received HDC and stem cell transplant before docetaxel; two achieved PR and one SD. Of the five patients who did not receive prior HDC, three achieved PR and two SD.

### *Toxicity* (Table 2)

In all, 44 cycles of docetaxel were administered to 13 patients; 42 cycles are assessable for haematological toxicity.

Neutropenia was the commonest haematological toxicity. Nadir neutropenia was early, in 33 out of 35 cycles (94%) it occurred on day 7. Grades III and IV neutropenia were observed in 15 and 53%, respectively, of docetaxel cycles in patients who had received previous HDC, and in 48 and 17% of cycles in those who had not received previous HDC. Recovery of neutrophil count to >1×10^9^ l^−1^ occurred in all cases within 1 week. There was only one episode of neutropenic sepsis. Toxicity-related (neutropenic sepsis) dose reduction occurred in only one patient. Treatment was delayed in only one patient (for 1 week) owing to grade I thrombocytopenia.

Most nonhaematological toxicities were acceptable and easily manageable, that is, Grades I and II Table 2

**Table 2:** Haematological and nonhaematological toxicities in relation to number of assessable docetaxel cycles

**Toxicity** (**no. of cycles**)	**Grade I (%)**	**Grade II (%)**	**Grade III (%)**	**Grade IV (%)**
Haemoglobin (42)	—	9 (21)	—	—
Neutrophils (42)	2 (5)	5 (11)	14 (33)	14 (33)
Platelets (42)	3 (7)	1 (2)	—	1 (2)
Liver chemistry (42)	4 (10)	1 (2)	1 (2)	—
Nausea/Vomiting (44)	3 (7)	—	—	—
Constipation (44)	10 (22)	—	—	—
Diarrhoea (44)	4 (9)	—	—	—
Stomatitis (44)	1 (2)	4 (9)	—	—
Neuropathy (44)	3 (7)	—	—	—
Fluid retention (44)	3 (7)	2 (5)	—	—
Skin (44)	3 (7)	2 (5)	1 (2)	—

. One patient developed grade III desquamating skin rash which precluded her for continuing treatment despite having achieved a PR.

## DISCUSSION

Docetaxel in this group of heavily pretreated poor prognosis patients demonstrated PR in 42% of patients after two cycles. However, responses were short lived; only one patient maintained such response until after the end of full treatment. Budman *et al* (1997) reports 13% major response (CR+PR) and 47% overall response (CR+PR+SD) in 55 evaluable patients with low and intermediate grade NHL treated with docetaxel without any steroid premedication and after only one or two prior chemotherapy regimens. In our study, dexamethasone was included in the regimen as premedication and antiemetic treatment. This may have contributed to the observed antitumour effect. However, such steroid treatment is considered a generally adopted practice.

Response to treatment did not appear to be influenced by disease type (HD/NHL), response to previous chemotherapy or disease status at trial entry. However, patients who had previous HDC appeared to respond less well to docetaxel. Previous studies using taxanes report a trend towards a better response in patients with relapsed as compared to refractory disease. In a phase II study, 68 patients with low and intermediate grades NHL who had either relapsed after or failed to respond to chemotherapy were treated by docetaxel. In low-grade NHL, all of the responses were seen in the relapsed patients. In intermediate grade NHL, a response was seen in one of 16 with refractory disease and in four of five with relapsed disease (Budman *et al*, 1997). In another phase II study evaluating paclitaxel in 96 patients with NHL, the response rates were higher in patients with relapsed than in those with refractory disease (37 *vs* 11%), respectively (Younes *et al*, 1997). In contrast, there was no suggestion of an association between the response rates to paclitaxel and the presence of chemotherapy resistant *vs* sensitive disease (22 *vs* 15%, respectively) in 31 patients with NHL (Wilson *et al*, 1995).

The median duration of response was 16 weeks (10–21). This is comparable to the response reported by Budman *et al* (1997) (1.4–20 months). Median survival in weeks decreased from 120 in responders, to 53 in SD and to 28 in SD and PD together indicating a poor outcome in nonresponders. However, one patient progressed after two cycles of docetaxel, but had a CR to subsequent chemotherapy regimen (ChlVPP) and is now alive and disease free.

After progression on docetaxel 10 out of 13 patients received a further one or two regimens of chemotherapy (including rituximab). One of them had HDC and peripheral blood stem cell rescue. Three out of 13 received radiotherapy with or without chemotherapy. Two patients received no further anticancer therapy.

El Helw *et al* (2001) investigated an anthracycline-based regimen; VEDex (vincristine, epirubicin, dexamethasone) in patients with NHL who were heavily pretreated or who had poor performance status. They reported 66.6% overall response rate and a median survival of 6 months. Shamash *et al* (2000) reviewed 37 patients with HD relapsing after HDC treated by a variety of single and combination chemotherapy. They report a median survival of 8 and 13.5 months, respectively. This compares to median survival of 35 (22–178) weeks for the four HD patients in our series (Three out of four received HDC followed by docetaxel). Other studies have used paclitaxel in NHL patients. Younes *et al* (1997) and Wilson *et al* (1995) reported response rates (CR+PR) of 25 and 27%, respectively. Younes *et al* (2001) also investigated the effectiveness of adding another cytotoxic agent (topotecan) to paclitaxel with 48% overall response rate.

Overall, treatment was well tolerated with the exception of one patient who developed intolerable skin toxicity. Although neutrophil toxicity was common, we had only one episode of neutropenic sepsis. Fossella *et al* (2000) compared the use of docetaxel 100 and 75 mg m^−2^ in previously treated nonsmall cell lung cancer patients with reduction of grade IV neutropenia from 77 to 54% and febrile neutropenia from 12 to 8% and recommended the use of 75 mg m^−2^. Reducing the dose to 70 mg m^−2^ in two of our patients prevented grade IV neutropenia in these patients.

## CONCLUSION

This small study suggests that the use of moderate dose docetaxel every 3 weeks for heavily pretreated patients with refractory or relapsed HD and NHL is feasible, safe and marginally effective. However, responses are not durable and routine use of single docetaxel in this setting cannot be recommended. Newer drugs and approaches are needed to be evaluated in this group of patients.
